# Sick pets as potential reservoirs of antibiotic-resistant bacteria in Singapore

**DOI:** 10.1186/s13756-018-0399-9

**Published:** 2018-08-31

**Authors:** Sri Harminda Pahm Hartantyo, Man Ling Chau, Laurent Fillon, Ahmad Zhafir Bin Mohamad Ariff, Joanne Su Lin Kang, Kyaw Thu Aung, Ramona Alikiiteaga Gutiérrez

**Affiliations:** 1 Environmental Health Institute, National Environment Agency, 11 Biopolis Way, #04-03/04/05, Helios Block, Singapore, 138667 Singapore; 2 Veterinary Emergency and Specialty Hospital Pte Ltd, 2-14 Rochdale Road, Singapore, 535815 Singapore; 3 School of Chemical and Biomedical Engineering, Nanyang Technological University, 62 Nanyang Drive, Singapore, 637459 Singapore

**Keywords:** Pets, Companion animals, Antimicrobial resistance, Antibiotic-resistant bacteria

## Abstract

An analysis of 186 diagnostic reports collected from a veterinary clinic in Singapore between 2014 to 2016 showed that sick companion animals can carry bacteria that are of significance to human health. Among the 186 specimens submitted, 82 showed polymicrobial growth (45%, 82/186) and in total, 359 bacteria were isolated. Of the 359 bacteria reported, 45% (162/359) were multi-drug resistant and 18% (66/359) were extended-spectrum-beta-lactamase species. Resistance to broad-spectrum antibiotics were also observed among individual species. Namely, methicillin-resistance among *Staphylococcus pseudintermedius* (63%, 32/51) and *Staphylococcus aureus* (50%, 4/8); fluoroquinolone-resistance among *Escherichia coli* (40%, 17/42) and carbapenem-resistance among *Klebsiella pneumoniae* (7%, 2/30) were noted. Our analysis suggests that sick pets may contribute to the pool of clinically relevant antibiotic-resistant bacteria and play a role in the spread of antibiotic resistance in Singapore. A more extensive study to better understand the extent of distribution and the factors affecting transmission of antibiotic-resistant bacteria to and from pets is necessary.

## To the editor

The development of antimicrobial resistance (AMR) in organisms common to food and companion animals (e.g. *Escherichia coli*, *Staphylococcus* sp., *Enterococcus* sp.) and among opportunistic pathogens (e.g. *Klebsiella* sp., *Acinetobacter* sp.) has been reported in several countries [1–3]. The transfer of antimicrobial-resistant bacteria from animals to humans is a pressing public health concern particularly when resistance is towards antibiotics used in human medicine [4].

We report a preliminary descriptive analysis investigating the presence of AMR in bacteria isolated from sick companion animals in Singapore. This analysis shall guide further efforts to better understand the contribution of pets to the pool of antibiotic-resistant organisms in the environment and community in Singapore.

One hundred eighty-six (186) veterinary diagnostic reports from a single, community-based private companion animal practice in Singapore, were retrospectively examined. Diagnostic reports, dated 2014 to 2016, contained bacteria isolation results and associated antibiograms of consecutive specimen submissions from pets presenting to the clinic. Bacteria and antimicrobial resistance screening tests were originally requested by veterinarians to guide diagnostic decisions when initial treatment of pathologies was ineffective. Specimens screened were from canine and feline species, and were collected from wounds/abscess (25%, 45/183); ear swabs (24%, 43/183); urine (20%, 37/183) and other samples such as skin and nasal swabs (32%, 58/183). Among these specimens, polymicrobial growth was observed in 53% (24/45) of wound/abscess, 58% (25/43) of ear swab, 22% (8/37) of urine, and 43% (25/58) of all other samples. While there were differences in testing panels, bacteria were screened for susceptibility to antibiotics belonging to at least four of the following antibiotic classes: aminoglycoside, carbapenem, carbolic acid, macrolide, cephalosporin, chloramphenicol, fluoroquinolone, penicillin, rifampin, sulfonamide and tetracycline. Interpretations of antimicrobial susceptibility were based on standard minimum inhibitory concentrations, as described by the Clinical and Laboratory Standards Institute [5].

**Fig. 1:**
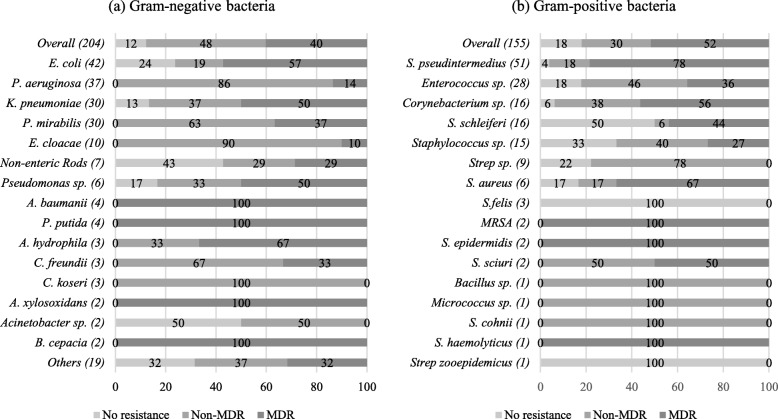
Antimicrobial resistance profiles of **a** gram-negative and **b** gram-positive bacteria isolated from sick pets. Numbers inside brackets ‘()’ indicate number of isolates detected; those on bars indicate percentage per organism; MDR- multi-drug resistant

Resistance to multiple antibiotic classes and to broad-spectrum antibiotics was observed among bacteria isolated from sick pets. Forty-five percent (45%, 162/359) of bacteria isolated were multi-drug resistant (MDR), i.e. resistant to at least 3 antibiotic classes [6]; 40% (144/359) were resistant to 1–2 antibiotic classes and 15% (54/359) conferred no resistance to the antibiotics tested. The antimicrobial resistance profiles obtained are illustrated in Fig. 1. Seventy-eight percent (78%, 40/51) of *Staphylococcus pseudintermedius*, the most frequently detected bacteria (14%, 51/359), were MDR. Fifty-seven percent (57%, 24/42) of *E. coli* were MDR, and resistance to at least one fluoroquinolone antibiotic (ciprofloxacin, enrofloxacin, marbofloxacin, norfloxacin, ofloxacin) was observed for 40% (17/42) of them. A 50% (15/30) MDR prevalence rate was observed among *Klebsiella pneumoniae*, and 7% (2/30) showed resistance to carbapenem (imipenem). Multi-drug resistance was also noted in 44% (7/17) of *Staphylococcus schleiferi* (44%, 7/16) and in 56% (9/16) of *Corynebacterium* sp.. Among Gram-negative bacteria detected, 32% (66/204) were extended-spectrum-beta-lactamase (ESBL) organisms, as indicated by resistance to 1 or more 3rd-generation cephalosporins (cefotaxime, cefpodoxime, ceftazidime, ceftiofur or ceftizoxime). Forty-seven percent (47%, 14/30) of *K. pneumoniae* and 40% (17/42) of *E. coli* were ESBLs. Methicillin-resistance was observed among Gram-positive bacteria, particularly in *Staphylococcus* spp.. Based on resistance to oxacillin, 50% (4/8) of *S. aureus* and 63% (32/51) of *S. pseudintermedius* isolated from sick pets were methicillin-resistant.

Our findings show that bacteria of public health relevance – MDR species, ESBLs, methicillin-resistant *S. pseudintermedius* (MRSP) and *S. aureus* (MRSA) – could be found among sick pets. The detection of these clinically-relevant bacteria suggests that sick pets, as they live in close proximity with their owners, may play a role in the transmission of AMR between animal and human sources. Conversely, pet owners may also play a role in this transmission. Antimicrobial resistance in bacteria may not necessarily indicate increased pathogenicity, but the presence of MDR organisms in pets, or in any other sources, may affect the resistome and increase the chances of AMR spreading to other bacteria, through lateral gene transfer. Several studies have suggested that commensals, such as the *Corynebacterium* sp. noted in this analysis, may harbor resistance genes [1, 2, 7] and transfer antimicrobial resistant determinants to opportunistic pathogens [8]. Added measures, such as tighter restrictions to access higher-generation antibiotics, and educational efforts to increase awareness on the prudent use of such antibiotics, may be needed to curtail the spread of AMR in Singapore. Indeed, serious therapeutic challenges may arise if human infections occur due to bacteria that are able to resist the inhibiting effects of broad-spectrum antibiotics (i.e. ESBLs, fluoroquinolone-resistant *E. coli* and carbapenem-resistant *K. pneumoniae*). Carbapenem resistance in particular, suggests resistance to nearly all beta-lactam antibiotics commonly used for clinical treatment of gram-negative bacteria [9, 10]. In addition, the transmission of methicillin-resistant *S. pseudintermedius* to humans, albeit rare, has been increasingly reported by studies citing contact with pets as the likely source of infection [11–13]; pets have likewise been reported to become colonized by MRSA through human contact, then becoming a possible source of re-infection or recurrent MRSA infection for humans [14]. The detection of MDR *S. schleiferi* was also notable as this bacteria has been reported to cause both veterinary and human skin infections [15].

This initial assessment is confined to data obtained from retrospective veterinary diagnostic reports. These reports did not indicate whether the isolated bacteria were the causative agents of infection. Information on specimens such as faeces, that are not commonly sent for antibiotic resistance screening, were unavailable. Data on the pet’s prior antibiotic use, veterinary care or interaction with human carriers (if any) that may have increased chances of exposure to antibiotic-resistant bacteria were also unavailable from the diagnostic reports. Nevertheless, our analysis suggests that sick pets may contribute to the pool of clinically relevant antibiotic-resistant bacteria in Singapore and that a more extensive epidemiological study on AMR bacteria carried by sick pets is necessary to better understand the extent of distribution, the direction and risk factors pertaining to transmission as well as the associations between veterinary, environmental and clinical AMR data.

## Abbreviations

AMR: Antimicrobial resistance
ESBL: Extended-spectrum-beta-lactamase
MDR: Multi-drug resistant
MRSA: Methicillin-resistant *Staphylococcus aureus*
MRSP: Methicillin-resistant *Staphylococcus pseudintermedius*
